# Application of a Novel Anti-Adhesive Membrane, E8002, in a Rat Laminectomy Model

**DOI:** 10.3390/ijms19051513

**Published:** 2018-05-18

**Authors:** Kiyoshi Kikuchi, Kentaro Setoyama, Takuto Terashi, Megumi Sumizono, Salunya Tancharoen, Shotaro Otsuka, Seiya Takada, Kazuki Nakanishi, Koki Ueda, Harutoshi Sakakima, Ko-ichi Kawahara, Ikuro Maruyama, Gohsuke Hattori, Motohiro Morioka, Eiichiro Tanaka, Hisaaki Uchikado

**Affiliations:** 1 Division of Brain Science, Department of Physiology, Kurume University School of Medicine, 67 Asahi-machi, Kurume, Fukuoka 830-0011, Japan; kikuchi_kiyoshi@kurume-u.ac.jp; 2 Department of Neurosurgery, Kurume University School of Medicine, 67 Asahi-machi, Kurume 830-0011, Japan; hattori_gohsuke@kurume-u.ac.jp (G.H.); mmorioka@med.kurume-u.ac.jp (M.M.); 3 Department of Systems Biology in Thromboregulation, Kagoshima University Graduate School of Medical and Dental Sciences, 8-35-1 Sakuragaoka, Kagoshima 890-8520, Japan; koichi.kawahara@oit.ac.jp (K.K.); maruyama@m2.kufm.kagoshima-u.ac.jp (I.M.); 4 Department of Pharmacology, Faculty of Dentistry, Mahidol University, 6 Yothe Road, Rajthevee, Bangkok 10400, Thailand; salunya.tan@mahidol.edu; 5 Division of Laboratory Animal Science, Natural Science Center for Research and Education, Kagoshima University, 8-35-1 Sakuragaoka, Kagoshima 890-8520, Japan; seto@m.kufm.kagoshima-u.ac.jp; 6 Course of Physical Therapy, School of Health Sciences, Faculty of Medicine, Kagoshima University, 8-35-1 Sakuragaoka, Kagoshima 890-8544, Japan; k7200686@kadai.jp (T.T.); k9380225@kadai.jp (M.S.); k3360022@kadai.jp (S.O.); k5082701@kadai.jp (S.T.); k9378361@kadai.jp (K.N.); k1238698@kadai.jp (K.U.); sakaki@health.nop.kagoshima-u.ac.jp (H.S.); 7 Laboratory of Functional Foods, Department of Biomedical Engineering Osaka Institute of Technology, 5-16-1 Omiya, Asahi-ku, Osaka 535-8585, Japan; 8 Uchikado Neuro-Spine Clinic, 1-2-3 Naka, Hakata-ku, Fukuoka 812-0893, Japan

**Keywords:** failed back surgery syndrome, anti-adhesive membrane, E8002, laminectomy

## Abstract

Neuropathic pain after spinal surgery, so-called failed back surgery syndrome, is a frequently observed common complication. One cause of the pain is scar tissue formation, observed as post-surgical epidural adhesions. These adhesions may compress surrounding spinal nerves, resulting in pain, even after successful spinal surgery. E8002 is an anti-adhesive membrane. In Japan, a clinical trial of E8002 is currently ongoing in patients undergoing abdominal surgery. However, animal experiments have not been performed for E8002 in spinal surgery. We assessed the anti-adhesive effect of E8002 in a rat laminectomy model. The dura matter was covered with an E8002 membrane or left uncovered as a control. Neurological evaluations and histopathological findings were compared at six weeks postoperatively. Histopathological analyses were performed by hematoxylin–eosin and aldehyde fuchsin-Masson Goldner staining. Three assessment areas were selected at the middle and margins of the laminectomy sites, and the numbers of fibroblasts and inflammatory cells were counted. Blinded histopathological evaluation revealed that adhesions and scar formation were reduced in the E8002 group compared with the control group. The E8002 group had significantly lower numbers of fibroblasts and inflammatory cells than the control group. The present results indicate that E8002 can prevent epidural scar adhesions after laminectomy.

## 1. Introduction

Spinal surgery typically induces various degrees of scar tissue and adhesion formation in the epidural space, termed epidural fibrosis, and this fibrosis may cause problems if further surgery is required [1]. Epidural fibrosis can compress the intraspinal nervous tissues to induce a variety of symptoms including significant functional disability and recurrent radicular pain, and the resulting syndrome, failed back surgery syndrome (FBSS), was reported to affect 8–40% of patients undergoing lumbar disc surgery [2]. There are no effective treatments for patients with established epidural fibrosis, and the associated complications make revision surgery more complex and time-consuming, with most reoperations for FBSS being unsuccessful [3]. Prevention of epidural fibrosis formation is considered to be the best approach for FBSS [4], and various attempts have been undertaken toward such prevention.

Many biological and synthetic materials, including polymethyl methacrylate, polylactic acid, autologous leather, silastic silicone, and fat grafts, have been reported to show anti-fibrotic effects [5,6,7,8,9]. Pharmaceutical agents, such as mitomycin C, doxycycline, rapamycin, hydroxycamptothecine, colchicine, steroid hormone, and anti-inflammatory agents, have been used to reduce epidural fibrosis [10,11,12,13,14]. However, limited or variable success was achieved, and some of these medicines caused side effects such as wound infections. Therefore, it remains clinically urgent to develop new methods that can reduce epidural fibrosis.

E8002 is an anti-adhesive membrane that was previously known as nDM-14R [15]. E8002 is designed to have a three-layered structure. The central layer is composed of pullulans, which are used in foods and drugs, and are known to be innocuous and bioabsorbable. The surface layers are composed of l-lactide, glycolide, and ε-caprolactone copolymers (Taki Chemical, Kakogawa, Japan) produced by ring-opening polymerization catalyzed by tin octanoate [Sn(O_2_C_8_H_15_)_2_]. These polymers are also used in bioabsorbable sutures. The material used for the central layer readily dissolves under moist conditions, while the materials used for the surface layers are nearly insoluble. The thickness of the central layer is set at 30 μm, while the surface layers are approximately 100 nm. In Japan, a clinical trial on E8002 in patients undergoing abdominal surgery was initiated in 2007 and is currently ongoing [16]. However, animal experiments have not yet been performed for E8002 in spinal surgery. In the present study, we evaluated the therapeutic effect of local E8002 application on reduction of epidural fibrosis in a rat laminectomy model. If E8002 can inhibit fibroblast proliferation and reduce epidural fibrosis after laminectomy, this product may be effective for FBSS. The present results may provide a novel method for reducing epidural fibrosis, and may be applicable to future human trials on clinical use of E8002 for spinal surgery.

## 2. Results

### 2.1. E8002 Does Not Cause Neurological Adverse Effects

**Figure 1:**
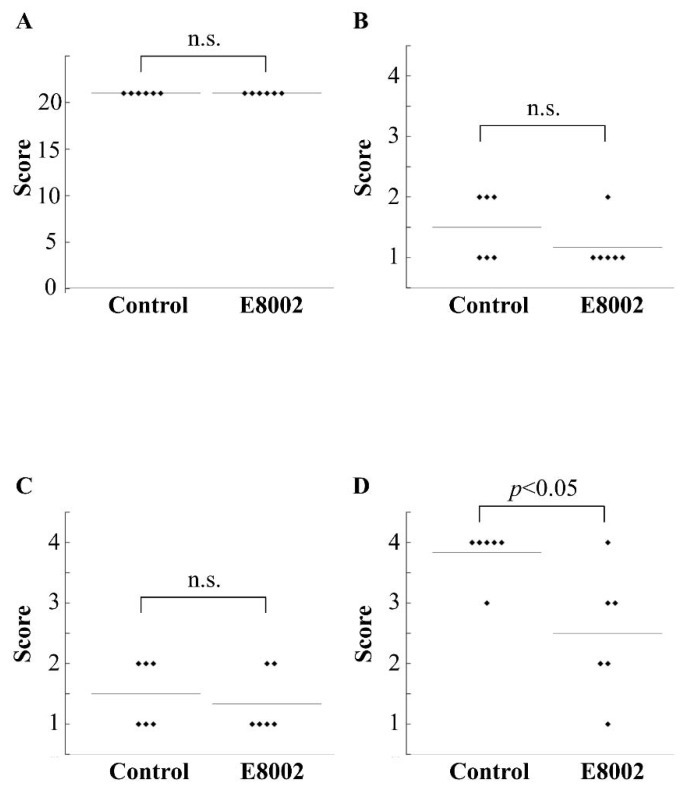
Effect of E8002 on neurological and macroscopic evaluations. (**A**) BBB score; (**B**) Macroscopic evaluation of skin; (**C**) Macroscopic evaluation of fascia; (**D**) Macroscopic evaluation of muscle. n.s., not significant.

We evaluated neurological adverse effects in the rat laminectomy model. Preoperative and postoperative comparisons of posture, weight support, and coordination according to the Basso, Beattie, and Bresnahan (BBB) locomotion test did not show significant changes between the E8002 group and the control group (BBB score = 21) (Figure 1A). These results confirmed that the nervous tissues were not injured intraoperatively by the properties of the inserted E8002 membrane. None of the rats died intraoperatively or postoperatively, and no obvious adverse effects were observed. Therefore, E8002 did not cause neurological adverse effects.

### 2.2. E8002 Induces Muscle Healing

The data from the macroscopic evaluations are shown in Figure 1B–D. The evaluations did not show significant differences in the skin and fascia between the two groups. However, muscle healing was significantly improved in the E8002 group compared with the control group.

### 2.3. E8002 Reduce Fibroblasts and Inflammatory Cells in Epidural Scar Tissues

**Figure 2:**
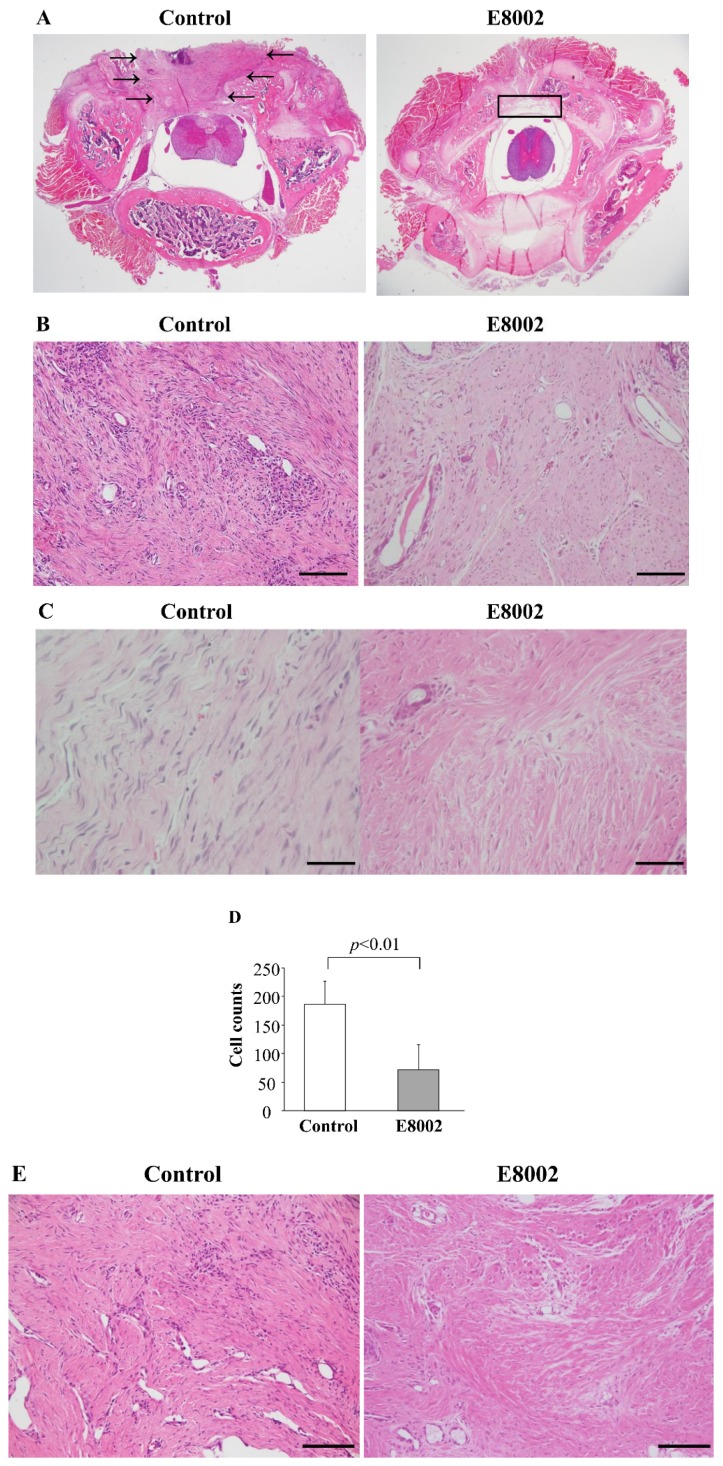

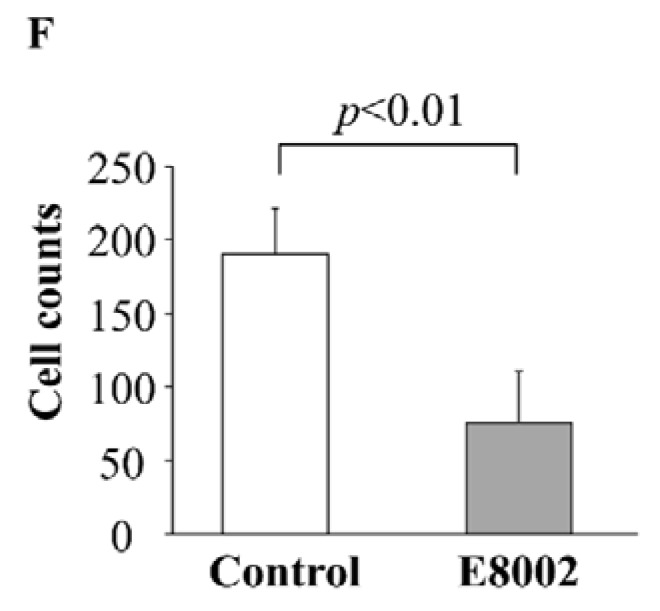
Effect of E8002 on epidural scar tissues evaluated by HE staining. (**A**) Photomicrographs of epidural adhesions at the laminectomy sites. Arrows indicate scar tissue. The square indicates vacuolation above the dura mater; (**B**) Representative images of HE staining for fibroblasts and inflammatory cells in the control group and E8002 group (original magnification, ×200). Scale bars, 100 μm; (**C**) Representative images of HE staining for fibroblasts in the control group and E8002 group (original magnification, ×400). Scale bars, 100 μm; (**D**) Counts of fibroblast density; (**E**) Representative images of HE staining for inflammatory cells in the control group and E8002 group (original magnification, ×400). Scale bars, 100 μm; (**F**) Cell counts of inflammatory cells.

**Figure 3:**
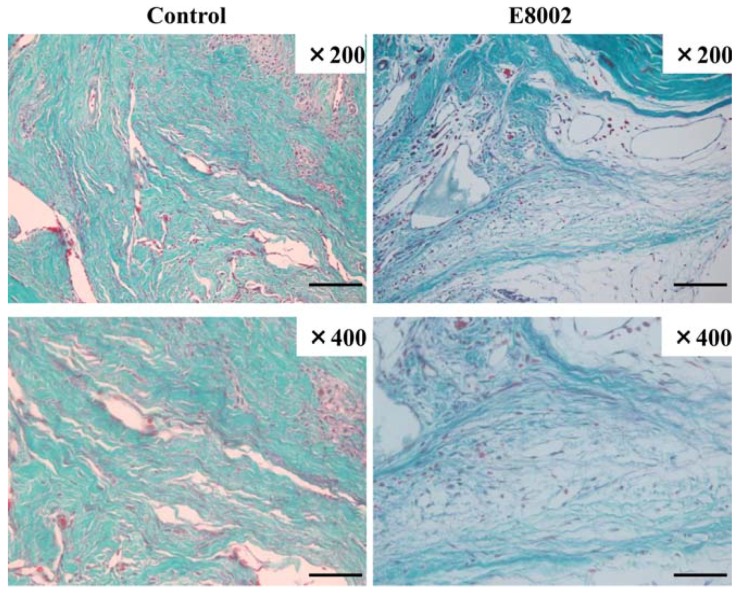
Effect of E8002 on epidural scar tissues evaluated by aldehyde fuchsin-Masson Goldner staining. Representative images of aldehyde fuchsin-Masson Goldner staining in the control group and E8002 group are shown (original magnification: upper panels ×200, lower panels ×400) (scale bars: **upper panels** 100 μm, **lower panels** 50 μm).

Typical images of hematoxylin–eosin (HE) staining of epidural scar tissues at the L1–L2 levels are shown in Figure 2A. In the control group, dense epidural scar tissue and compact collagen tissues were found at the laminectomy sites, and the scar tissue was widely adhered to the dura mater. In the E8002 group, vacuolation above the dura mater, loose scar adhesion, and less collagen tissues were observed. On histological examination, the fibroblast and inflammatory cell densities in the E8002 group were significantly lower than those in the control group (Figure 2B–F). Aldehyde fuchsin-Masson Goldner staining revealed that the E8002 group had fewer observable epidural scar adhesions compared with the control group (Figure 3).

## 3. Discussion

The present findings have demonstrated reductions in scar formation and adhesions after experimental laminectomy with E8002 treatment in a rat model.

Wound healing is generally a positive physiological event that restores the anatomy and function of tissues after injury, and the ideal end result is tissue restoration to the condition before the injury [13]. An important part of the wound healing process is the formation of connective tissue or scar tissue that supports the healing tissues during regeneration [13]. However, in many cases, the newly formed connective tissue (scar tissue) can negatively interfere with the normal function of the healing tissues [13]. Following abdominal and gynecologic surgery, it is not uncommon for the surgical procedure per se to induce adhesions that not only make subsequent surgery more difficult, but also lead to pathological conditions such as ileus or infertility [17]. Spinal surgery often results in dense scar formation termed epidural fibrosis. In some cases, this fibrosis induces significant difficulties for repeated surgery and has been reported to induce compression of the adjacent nerve tissue [1,2]. Epidural fibrosis is a major cause of FBSS. A method for controlling wound healing, particularly the formation of scar tissue and adhesions, would be of great value for post-surgical wound healing in most cases.

Many attempts have been undertaken to control scar formation. There are several methods that rely on barriers with various properties, and substantial numbers of biological and synthetic materials and pharmaceutical agents have been applied [5,6,7,8,9,10,11,12,13,14]. However, the results have not been entirely satisfactory. Because fibroblasts are responsible for producing collagen, much attention has been drawn to the regulation of fibroblasts to reduce scar formation [18,19,20]. In previous studies, certain compounds such as rapamycin, mitomycin C, and *all-trans* retinoic acid were shown to exert anti-adhesive effects by inhibiting fibroblast proliferation [11,12,14], similar to the case for E8002. However, there are no compounds with anti-adhesive effects that are widely used in the field of spinal surgery at clinical sites worldwide. A clinical trial using E8002 is currently ongoing in patients undergoing covering colostomy and colostomy closure. However, the spaces surrounding the intra-abdominal organs and those surrounding the spinal cord may be different. Nevertheless, even if the types of organs are different, the targets for adhesion prevention may be the same. Barriers between the two surfaces are considered the most effective method for preventing postoperative adhesions. For membrane-like anti-adhesive agents to exert an effect, two conditions are required: the damaged surface of all organs must be covered by the anti-adhesive agent until the early fibrin network is completed, and inflammatory cells must not invade the first fibrin network. The results of the above clinical trial may accelerate the start of clinical trials in patients undergoing spinal surgery.

In conclusion, the results of the present study suggest that E8002 can reduce scar formation/adhesions after spinal surgery. Although the underlying mechanisms remain to be clarified in further studies, the findings suggest the possibility for future design of potent pharmacological treatment modalities that can reduce post-surgical adhesion formation and scarring, potentially in combination with physical barriers. We should remain optimistic about the future of spinal surgery, and continue to explore new strategies to provide optimal care for patients undergoing spinal surgery.

## 4. Materials and Methods

The experimental protocol was approved by the Institutional Animal Care and Use Committee of Kagoshima University (Kagoshima, Japan). The study protocol was approved by the local ethics committee of Kagoshima University (Ethic approval number: MD17014, Approval date: 26 May 2017).

### 4.1. Rat Model of Laminectomy

A rat laminectomy model was used to determine the effects of E8002 on epidural fibrosis. A total of 12 male Sprague-Dawley rats aged 8 weeks and weighing 290–310 g were used in the study. Anesthesia was induced and maintained with 2.5–3.0% isoflurane inhalation and the animals were fixed in the prone position. The back hair at the L1–L2 level was shaved, and the skin was sterilized with iodophor three times. The laminectomy model was constructed as previously described [14]. All procedures were performed under sterile conditions with basic surgical tools, surgical microscopes, and an electrical drill. A median incision of the dorsal skin was made at the L1–L2 level, and the paraspinal muscles were separated. A rongeur was used to remove the spinous process and lamina, and the dura mater at the L1–L2 level was exposed. An E8002 membrane (3 × 2 mm) was applied to the surgical site. No membrane was applied in control rats. The total 12 rats were divided into two groups: control group (*n* = 6) and E8002 group (*n* = 6). Satisfactory hemostasis was achieved using gauze; bone wax and cauterization after laminectomy were not needed. All procedures were performed with care to avoid injury to the neural tissues.

### 4.2. Neurological Evaluation

At 6 weeks postoperatively, neurological evaluations were performed to confirm that the membrane did not prevent healing of the spinal cord and dura matter, or injure the nerve roots and spinal cord. All rats underwent preoperative and postoperative neurobehavioral assessments using the BBB locomotion test [21]. This test assessed posture, weight support, and coordination during open field locomotion.

### 4.3. Macroscopic Evaluation

After 6 weeks, the rats were re-anaesthetized. The skin and muscle wound healing was macroscopically graded by a person blinded to the experimental groups using a semiquantitative scale based on the Olmarker classification (healing of skin incision: 1, good healing; 2, slight diastasis; 3, pronounced diastasis; 4, infection; healing of fascia and muscle: 1, good healing; 2, slight diastasis; 3, clear diastasis; 4, hematoma or infection with loss of contact) as described [13], with small modifications.

### 4.4. Histological Analysis

At 6 weeks postoperatively, the rats were deeply anesthetized by intraperitoneal injection of pentobarbital sodium (100 mg/kg), and perfused with heparin physiological saline, followed by 4% paraformaldehyde in 0.1 M phosphate buffer (pH 7.4) via the heart. The entire L1–L2 vertebral column, including the paraspinal muscles and epidural scar tissue, was resected en bloc. The samples were fixed in 4% paraformaldehyde at 4 °C overnight, decalcified in Kalkitox (Wako Pure Chemical Industries Ltd., Osaka, Japan) at 4 °C for 2 days and 5% sodium sulfate solution at 4 °C overnight, dehydrated in a graded ethanol series, cleared with xylene, and embedded in paraffin. The paraffin-embedded samples were cut into 4-mm transverse sections through the L1–L2 vertebrae, and stained with HE. Aldehyde fuchsin-Masson Goldner staining was also performed to identify connective tissues, such as elastic fibers and collagen fibers. Epidural scar adhesions were evaluated under a light microscope (DP21; Olympus Optical Co., Tokyo, Japan). Three areas were selected at the center and margins of the laminectomy sites. The numbers of fibroblasts and inflammatory cells were counted in these three areas, and the mean value was calculated per ×400 field as previously described [22].

### 4.5. Statistical Analysis

Variability of data was assessed by the F-test for parametric data. Student’s *t*-test for independent samples was applied to determine the statistical significance of differences between the mean values of two study groups. Values of *p* < 0.05 were considered statistically significant. If data were not normally distributed, the Mann–Whitney-*U*-test was used. Values were presented as mean ± standard deviation (SD). All statistical analyses were performed with SPSS version 24 (IBM, Chicago, IL, USA).

## 5. Study Limitations

For surgeons, the most important factor in evaluating adhesions is not the histological change of the operative field, but the feeling when actually touching the field. Using the Rydell classification (grade 0, epidural scar tissue is not adherent to the dura mater; grade 1, epidural scar tissue is adherent to the dura mater, but easily dissected; grade 2, epidural scar tissue is adherent to the dura mater and difficult to dissect without disrupting the dura mater; grade 3, epidural scar tissue is firmly adherent to the dura mater and cannot be dissected) as previously described [23], we tried to perform evaluations correctly for neurosurgeons and veterinary surgeons. However, the blinded macroscopic evaluation was unable to reveal differences in adhesions and scar formation between the two groups. The fundamental factor for this is that the surgical field is very narrow in rats. Therefore, larger animal models may be needed to allow correct evaluations.

## Abbreviations

BBB	Basso, Beattie, and Bresnahan
FBSS	failed back surgery syndrome
